# UQCRH downregulation promotes Warburg effect in renal cell carcinoma cells

**DOI:** 10.1038/s41598-020-72107-2

**Published:** 2020-09-14

**Authors:** Yanting Luo, Louise Medina Bengtsson, Xuechun Wang, Tianhe Huang, Guoqiang Liu, Sean Murphy, Caiqin Wang, John Koren, Zachary Schafer, Xin Lu

**Affiliations:** 1 Department of Biological Sciences, Boler-Parseghian Center for Rare and Neglected Diseases, University of Notre Dame, 193 Galvin Life Sciences Center, Notre Dame, IN 46556 USA; 2 Harper Cancer Research Institute, University of Notre Dame, Notre Dame, IN 46556 USA; 3 Integrated Biomedical Sciences Graduate Program, Warren Family Research Center for Drug Discovery and Development, University of Notre Dame, Notre Dame, IN 46556 USA; 4 Department of Chemistry and Biochemistry, University of Notre Dame, Notre Dame, IN 46556 USA; 5 Tumor Microenvironment and Metastasis Program, Bren Simon Cancer Center, Indiana University Melvin, Indianapolis, IN 46202 USA

**Keywords:** Cancer, Cancer metabolism, Tumour-suppressor proteins, Urological cancer

## Abstract

Ubiquinol-cytochrome c reductase hinge protein (UQCRH) is the hinge protein for the multi-subunit complex III of the mitochondrial electron transport chain and is involved in the electron transfer reaction between cytochrome c1 and c. Recent genome-wide transcriptomic and epigenomic profiling of clear cell renal cell carcinoma (ccRCC) by The Cancer Genome Atlas (TCGA) identified *UQCRH* as the top-ranked gene showing inverse correlation between DNA hypermethylation and mRNA downregulation. The function and underlying mechanism of UQCRH in the Warburg effect metabolism of ccRCC have not been characterized. Here, we verified the clinical association of low UQCRH expression and shorter survival of ccRCC patients through in silico analysis and identified KMRC2 as a highly relevant ccRCC cell line that displays hypermethylation-induced UQCRH extinction. Ectopic overexpression of UQCRH in KMRC2 restored mitochondrial membrane potential, increased oxygen consumption, and attenuated the Warburg effect at the cellular level. UQCRH overexpression in KMRC2 induced higher apoptosis and slowed down in vitro and in vivo tumor growth. UQCRH knockout by CRISPR/Cas9 had little impact on the metabolism and proliferation of 786O ccRCC cell line, suggesting the dispensable role of UQCRH in cells that have entered a Warburg-like state through other mechanisms. Together, our study suggests that loss of UQCRH expression by hypermethylation may promote kidney carcinogenesis through exacerbating the functional decline of mitochondria thus reinforcing the Warburg effect.

## Introduction

Metabolic reprograming is a crucial hallmark of cancer^1^ and is particularly revealing in clear cell renal cell carcinoma (ccRCC)^2–4^. ccRCC is the most common (**∼ **75%), lethal subtype of kidney cancer. Recurrent mutations of metabolism-regulating genes, such as *VHL, PTEN, MTOR, PI3CA* and *TSC1/2,* and broader epigenetic and post-translational dysregulation of components of various metabolic pathways lead to the concept that ccRCC is a metabolic disease^2,3^. Among the metabolic shifts, upregulation of aerobic glycolysis (the “Warburg effect”) and downregulation of the tricarboxylic acid (TCA) cycle and oxidative phosphorylation (OXPHOS) are evidenced by profiling of transcriptomics^5^, proteomics^6^ and metabolomics^7^. Isotype tracing experiment of human cancers demonstrated ccRCC as a tumor type with convincing Warburg effect, whereas two other tumor types tested (brain and lung) displayed preserved and even heightened activity of glucose oxidation^8^. Therefore, ccRCC appears to be metabolically unique and display much stronger Warburg effect and mitochondrial dysfunction than other solid tumors. It is important to identify the molecular mechanisms underlying the dysregulated mitochondrial function in ccRCC. Hypoxia-inducible factor 1 (HIF-1) was found to negatively regulate mitochondrial biogenesis and OXPHOS in *VHL*-defective ccRCC cells through MYC inhibition^9^. However, because HIF-1α and HIF-2α have opposing activity in ccRCC with HIF-2α playing the dominant tumor-promoting role^10–12^, it remains unclear if the VHL-HIF axis is sufficient to explain the downregulated mitochondrial function in ccRCC.

New insights are emerging from the recent TCGA (The Cancer Genome Atlas) study of ccRCC, which identified *ubiquinol-cytochrome c reductase hinge protein* (*UQCRH*) as the top-ranked gene with profound promoter hypermethylation and inverse correlation with the mRNA level^5^. The recent Clinical Proteomics Tumor Analysis Consortium (CPTAC) study on ccRCC further identified that UQCRH is among the 565 significantly downregulated proteins in ccRCC tumors relative to normal adjacent tissues^6^. UQCRH is an integral subunit (the hinge protein) of the 11-subunit complex III (the ubiquinol:cytochrome c oxidoreductase) of the electron transport chain (ETC) in the mitochondrion. In the ETC, complex III shunts electrons from ubiquinol to cytochrome c and concomitantly pumps protons from the mitochondrial matrix to the intermembrane space to participate in the establishment of the membrane potential^13^. Complex III also contributes to the production of reactive oxygen species (ROS)^14^. Biochemically, UQCRH functions as a regulatory protein for the electron transfer reaction between cytochrome c1 and c^15^. UQCRH does not appear to be an essential component of complex III, because yeast with the UQCRH homolog deleted was still capable of growth on a nonfermentable substrate, suggesting that UQCRH was not essential for the assembly and function of complex III^16,17^. Nevertheless, electron transport activity of complex III in mutant yeast was merely about 40% of the wild type yeast^17^.

The role of UQCRH in cancer is poorly understood. Hypermethylation-induced UQCRH downregulation was reported for ovarian and breast cancer cell lines^18^. Clinical association of UQCRH expression and cancer prognosis has been explored with conflicting results. Whereas UQCRH was significantly downregulated and correlated with higher stage, poorer survival, and early recurrence in ccRCC patients^19^, it was significantly upregulated in lung adenocarcinoma^20^ and hepatocellular carcinoma^21^. In the current study, we focused on the function of UQCRH in ccRCC using a ccRCC cell line, KMRC2, in which *UQCRH* is hypermethylated. We found that overexpression of UQCRH in KMRC2 reprogrammed the metabolic activity at several levels and demonstrated the metabolically-linked tumor suppressive activity of UQCRH in at least some of the ccRCC cases.

## Results

### UQCRH downregulation in ccRCC is associated with DNA hypermethylation and worse overall survival

**Figure 1:**
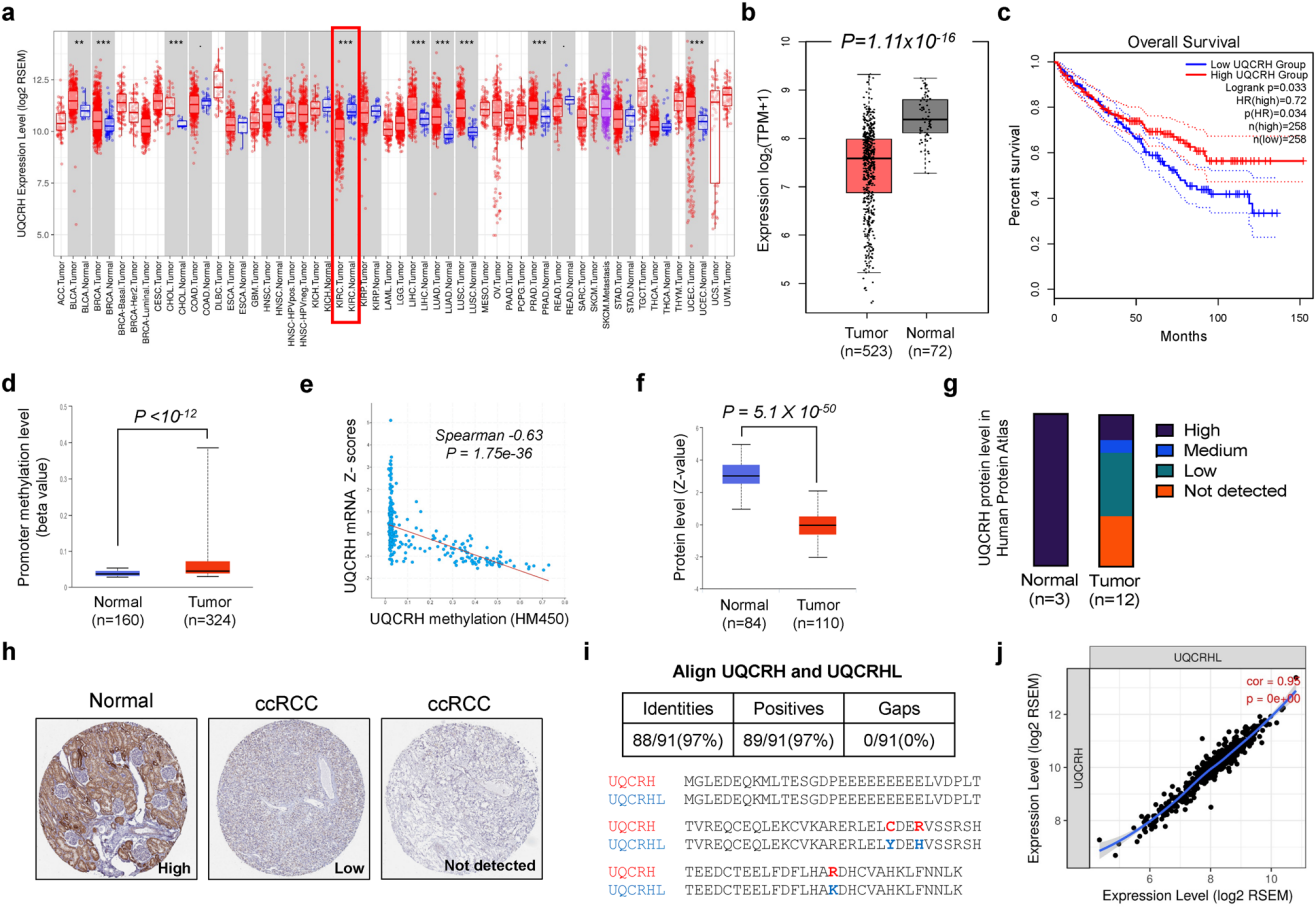
UQCRH is downregulated in ccRCC and associated with poor prognosis. **(a, b)** Significant downregulation of *UQCRH* mRNA level in ccRCC tumor samples compared with normal samples, based on TCGA dataset and analyzed using TIMER and GEPIA, respectively. **(c)** Significant association between lower *UQCRH* mRNA level and shorter overall survival in the TCGA ccRCC patients. The cutoff for low and high UQCRH expression was set at the midpoint of the total patient cohort by GEPIA. The calculation was based on Cox proportional-hazards model. **(d)** Significantly higher promoter methylation level of *UQCRH* in ccRCC tumor samples compared with normal samples, based on TCGA dataset and analyzed using UALCAN. **(e)** Significant inverse correlation between methylation and mRNA expression of *UQCRH* of the TCGA ccRCC tumor samples, analyzed by cBioPortal. **(f)** Significant downregulation of UQCRH protein level in the CPTAC ccRCC cohort, analyzed by UALCAN. **(g, h)** Summary and representative images of UQCRH immunohistochemistry staining intensities in normal kidney and ccRCC, based on Human Protein Atlas database. **(i)** Alignment of human UQCRH and UQCRHL protein sequences. **(j)** Tightly correlated expression of UQCRH and UQCRHL in the TCGA ccRCC tumors, analyzed with TIMER. In the plots above, cohort size and significance level (P value) are noted in the plots. When not specified, unpaired t-test was used.

To examine the expression pattern of *UQCRH* in cancers, we surveyed the TCGA data using two computational tools, TIMER and GEPIA, and observed that ccRCC was the only cancer type among various TCGA cancer types that showed significantly lower mRNA level of *UQCRH* in tumors compared with normal tissues (Fig. 1a, b). Importantly, lower UQCRH was associated with shorter overall survival (*P* = 0.033, Fig. 1c). In the TCGA ccRCC cohort, *UQCRH* promoter region was significantly hypermethylated (Fig. 1d), and the methylation was inversely correlated with mRNA expression (Fig. 1e). At the protein level, we used UALCAN to survey UQCRH expression in the CPTAC study where 110 treatment-naïve ccRCC tumors and 84 paired normal adjacent tissues were profiled^6^. There was a significant downregulation of UQCRH in the tumors (Fig. 1f), consistent with the retrieved tissue microarray staining result from the Human Protein Atlas (Fig. 1g, h). In both TCGA and CPTAC cohorts, besides the precipitous drop of UQCRH expression in ccRCC tumors relative to the normal tissue, the further correlation of UQCRH expression with tumor stages or grades appeared weak or absent by in silico analysis (Supplementary Fig. 1). Nonetheless, evidence exists to support that UQCRH expression decline is correlated with the disease progression in ccRCC^19^.

*UQCRHL* is the paralog of *UQCRH* and encodes a highly similar protein (97% identical, Fig. 1i). Both genes are located in chromosome 1p. To examine the possibility that UQCRHL compensates for the loss of UQCRH expression, we surveyed the ccRCC TCGA dataset and observed tightly correlated expression of these two genes (Fig. 1j). In fact, *UQCRHL* was the most correlated gene for *UQCRH*, suggesting that it is unlikely for UQCRHL to functionally compensate for UQCRH downregulation in the clinical samples.

### Ectopic overexpression and knockout of UQCRH in ccRCC cell lines

**Figure 2:**
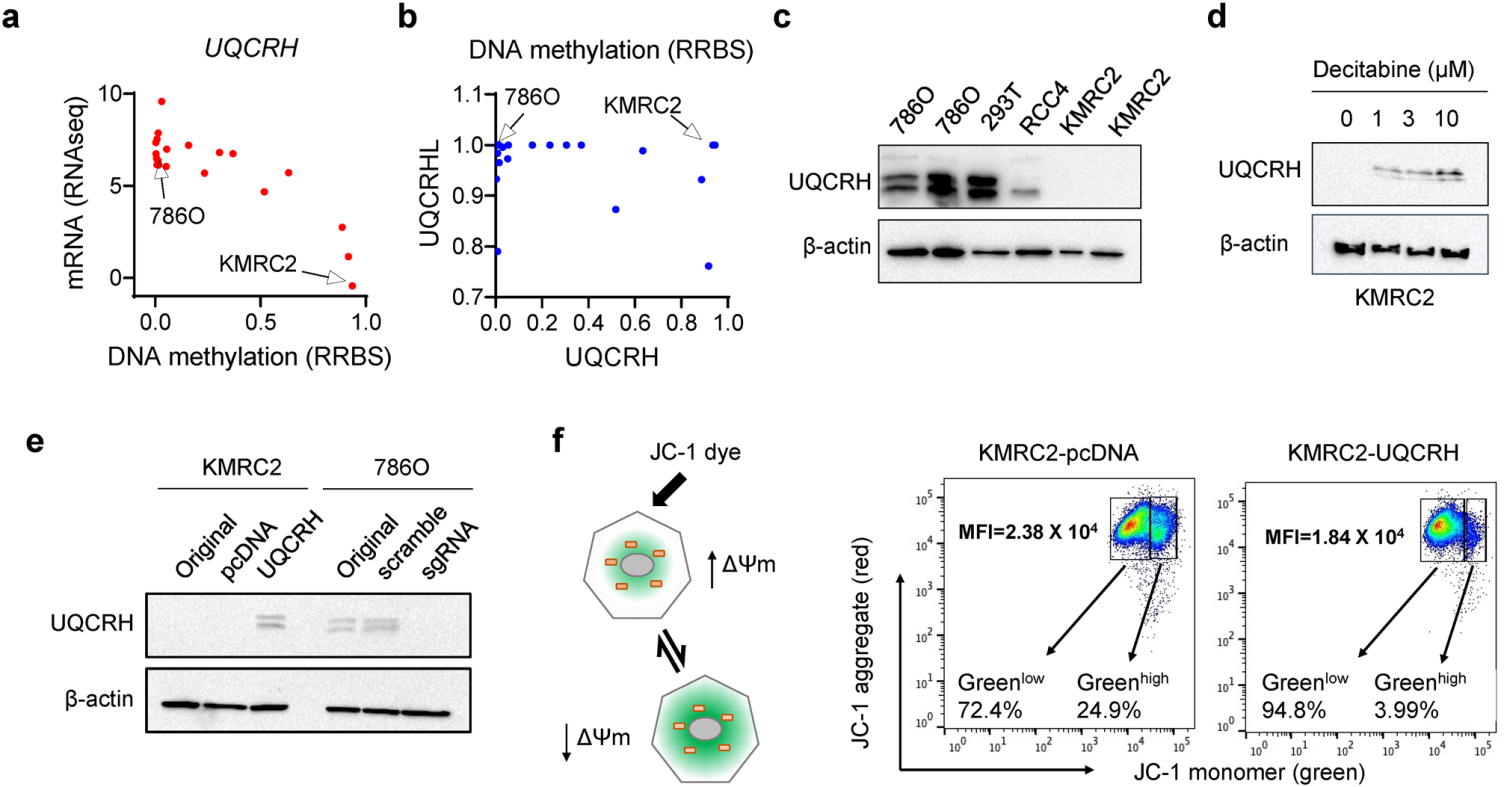
Restored UQCRH expression in KMRC2 repolarized mitochondrial membrane potential. **(a)** Inverse correlation of methylation and mRNA level of *UQCRH* in 21 ccRCC cell lines, data available from CCLE and drawn in Graphpad. **(b)** Methylation level of *UQCRH* and *UQCRHL* in 21 ccRCC cell lines, data available from CCLE and drawn in Graphpad. **(c)** UQCRH protein expression in 293T, 786O, RCC4 and KMRC2, detected by western blot. **(d)** Dose-dependent effect on UQCRH protein expression in KMRC2 by decitabine treatment, detected by western blot. **(e)** UQCRH ectopic overexpression in KMRC2 and UQCRH knockout by CRISPR/Cas9 in 786O, detected by western blot. **(f)** Principle and representative result of JC-1 assay with MFI of green fluorescence signals labeled. When mitochondrial membrane potential is disrupted, JC-1 cannot enter mitochondria effectively and remain as green fluorescent monomer in the cytosol.

To study the function of UQCRH in ccRCC, we surveyed the mRNA expression and promoter methylation pattern of *UQCRH* in 21 ccRCC cell lines with data available from the Cancer Cell Line Encyclopedia (CCLE) database^22^. Similar to the tumor samples, the mRNA level and DNA methylation pattern in ccRCC cell lines also exhibited an inverse correlation (Fig. 2a). Among the ccRCC lines, KMRC2^23^ showed very low UQCRH expression and very high methylation, whereas 786O showed the opposite pattern (Fig. 2a). KMRC2 also showed strong methylation for *UQCRHL* (Fig. 2b), suggesting that it is unlikely for UQCRHL to functionally compensate for the low UQCRH expression in KMRC2. UQCRH expression pattern was validated by western blot in ccRCC cell lines KMRC2, 786O and RCC4, with the human embryonic kidney cell line 293T as control (Fig. 2c).

We treated KMRC2 with decitabine, a DNA methyltransferase (DNMT) inhibitor, and observed a dose-dependent increase in UQCRH expression (Fig. 2d). This result verified that hypermethylation accounts for the silenced expression of UQCRH in KMRC2. We generated stable UQCRH ectopic overexpression in KMRC2 and CRISPR/Cas9 knockout of *UQCRH* in 786O (Fig. 2e). Next, we measured the effect of UQCRH overexpression on mitochondrial membrane potential (ΔΨm) using the JC-1 dye (Fig. 2f). Depolarization of ΔΨm by factors such as inadequate ETC function will lead to higher green fluorescence of JC-1. Indeed, we observed that UQCRH overexpression, presumably by rescuing complex III activity and repolarizing ΔΨm, rendered cells with much lower green fluorescence (Fig. 2f). This result indicates that UQCRH overexpression in KMRC2 improves the mitochondrial function.

### UQCRH overexpression partially overturns the Warburg effect

**Figure 3:**
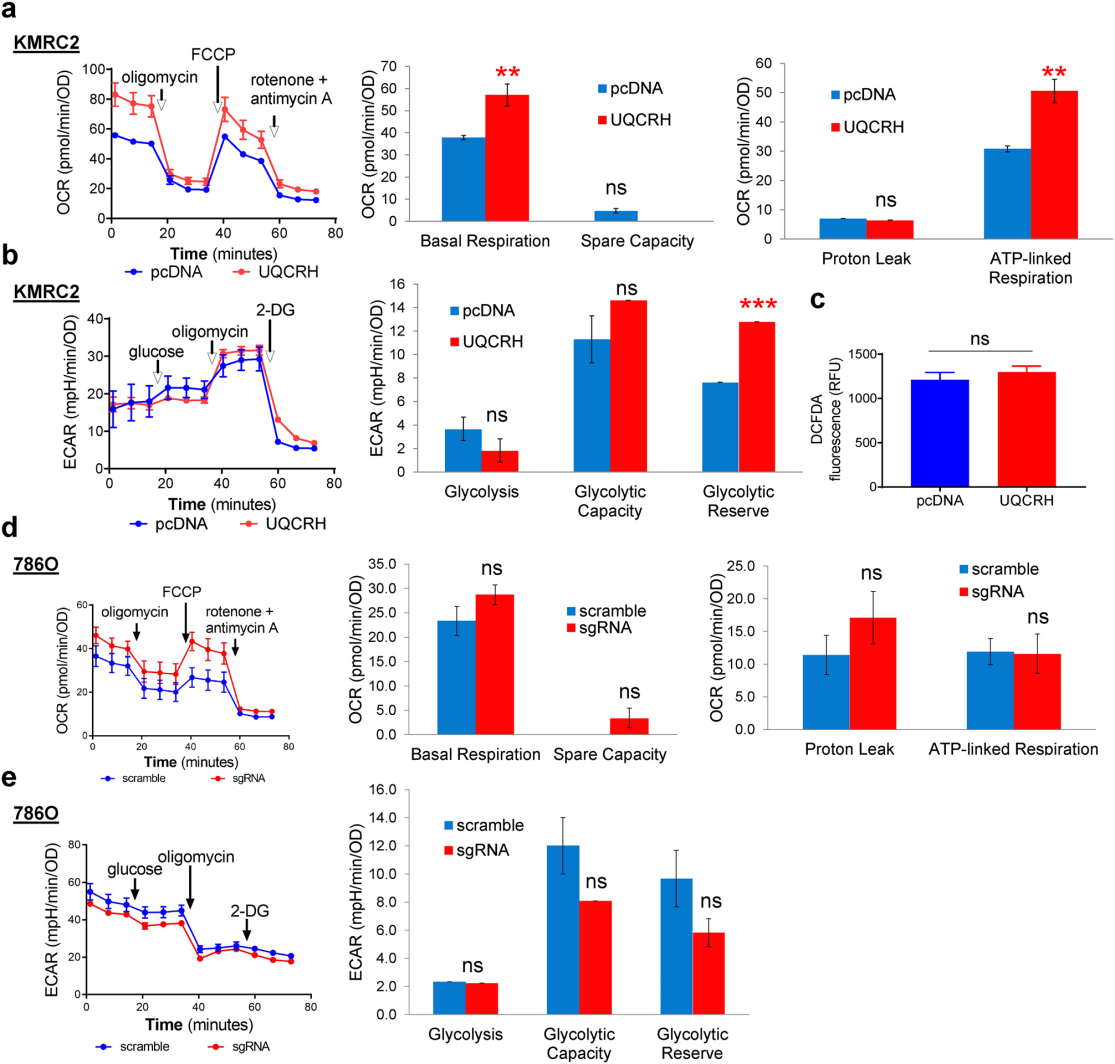
UQCRH overexpression partially overturns the Warburg effect in KMRC2. **(a)** UQCRH overexpression increased basal and ATP-linked oxygen consumption, detected by the Seahorse XF Mitochondrial Stress Test. **(b)** UQCRH overexpression increased glycolytic reserve, detected by the Seahorse XF Glycolytic Stress Test. **(c)** Unchanged intracellular ROS level in two KMRC2 sublines, detected by DCFDA assay. **(d–e)** Effect of *UQCRH* knockout in 786O on OCR in Mitochondrial Stress Test and ECAR in Glycolytic Stress Test. In all plots, data represent mean ± SD with n ≥ 4. ** P < 0.01, *** P < *0.001, *^*ns*^* P* > *0.05,* by unpaired t-test.

To understand how energy metabolism was affected by the level of UQCRH, we subjected KMRC2-pcDNA, KMRC2-UQCRH, 786O-scramble and 786O-sgRNA cells to Seahorse assays. Seahorse Extracellular Flux Analyzer measures glycolysis by analyzing extracellular acidification rate (ECAR) and measures mitochondrial OXPHOS based on oxygen consumption rate (OCR), through real-time and live cell analysis. To examine the effect of UQCRH overexpression on mitochondrial function, we used Mitochondrial Stress Test. Through measuring OCR at the basal level and in response to the serially added mitochondrial modulators (oligomycin, FCCP, and a mix of rotenone and antimycin A), mitochondrial respiration parameters can be calculated. In comparison to KMRC2-pcDNA, KMRC2-UQCRH showed significantly higher basal respiration and ATP-linked respiration (*P* < 0.01, Fig. 3a), consistent with the higher ΔΨm by UQCRH overexpression. In a somewhat unexpected manner, both KMRC2 sublines appeared to operate at their respective near-maximal respiration levels, because the FCCP-induced maximal respiration level was comparable to the basal respiration level (i.e. low spare capacity). This may be due to the inherently downregulated mitochondrial activity of this ccRCC cell line.

The Glycolysis Stress Test measures basal glycolysis upon addition of glucose and glycolysis capacity upon blockage of mitochondrial ATP production with oligomycin. Both KMRC2 sublines had similar non-glycolytic acidification before adding glucose (Fig. 3b). After adding glucose, ECAR increase was slightly lower for KMRC2-UQCRH relative to KMRC2-pcDNA (albeit not statistically significant), possibly due to the moderately enhanced ability of UQCRH^+^ cells to channel pyruvate (product of glycolysis) to the TCA cycle and OXPHOS instead of lactate. Upon oligomycin addition, KMRC2-UQCRH reached a slightly higher level in ECAR (albeit not statistically significant). Importantly, KMRC2-UQCRH exhibited a significantly higher reserved glycolytic potential relative to KMRC2-pcDNA (*P* < 0.001, Fig. 3b), suggesting that UQCRH overexpression reprogrammed the cells to rely less on glycolysis (thus the cells possess higher reserved glycolytic potential), because OXPHOS was sufficient to meet most of the energy needs. Lastly, cellular ROS level did not seem to be influenced by UQCRH status, as assessed by the oxidation of DCFDA (Fig. 3c). Collectively, these data indicate that UQCRH overexpression partially reverses the Warburg effect for KMRC2 cells.

When 786O-scramble and 786O-sgRNA were compared in Mitochondrial Stress and Glycolysis Stress tests, no significant differences were observed in the assessed parameters in either assay (Fig. 3d,e). This result could be caused by the fact that 786O cells already had very high glycolytic activity and severely weakened mitochondrial function, due to hyperactivated HIF signaling as a result of *VHL* mutation^9^. Therefore, UQCRH knockout would not further strengthen the Warburg-like metabolic activity in this cell line.

### UQCRH overexpression slows down ccRCC growth in vitro and in vivo

**Figure 4:**
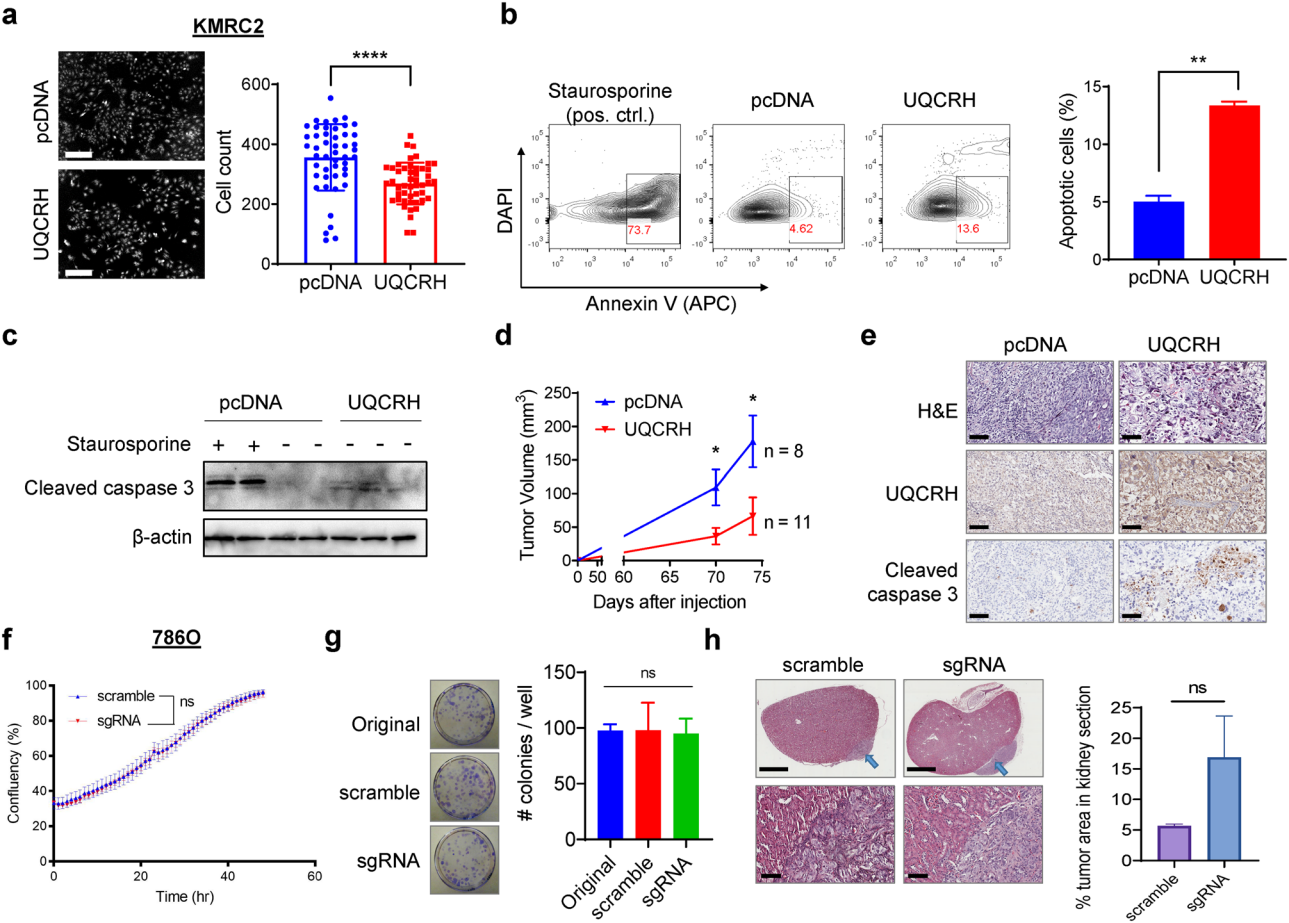
UQCRH overexpression reduces in vitro proliferation and in vivo tumor growth for KMRC2 cells. **(a)** Representative images of PI-stained nuclei and quantification result at the endpoint of the proliferation assay on the two KMRC2 sublines. Scale bar 300 μm. **(b)** Representative flow cytometry plots and quantification result of the apoptosis assay on two KMRC2 sublines. **(c)** Detection of cleaved caspase 3 in KMRC2-pcDNA and KMRC2-UQCRH by western blot. KMRC2-pcDNA treated with Staurosporine was used as positive control. **(d)** Tumor volumes in Rag1 knockout mice injected subcutaneously with KMRC2-pcDNA and KMRC2-UQCRH. **(e)** Representative images of H&E staining and IHC of UQCRH and cleaved caspase 3 in KMRC2-pcDNA and KMRC2-UQCRH tumors. Scale bar 100 μm. **(f)** IncuCyte confluency curves for 786O-scramble and 786O-sgRNA proliferation in vitro. **(g)** Representative images and quantification result of crystal violet-stained colonies formed by 786O-scramble and 786O-sgRNA (n ≥ 4). **(h)** Representative H&E images and percentage of tumor area relative to kidney area for orthotopic tumors formed by 786O-scramble and 786O-sgRNA (n = 2/group). Blue arrows point to the tumor area. Scale bar 1.5 mm (upper) and 100 μm (lower). In all plots, data represent mean ± SD. * P < 0.05, ** P < 0.01, **** P < *0.0001, *^*ns*^* P* > *0.05,* by Mann–Whitney test (for h) or unpaired t-test (for others).

Compared with KMRC2-pcDNA, the overall proliferation of KMRC2-UQCRH was significantly reduced (Fig. 4a). Cell apoptosis assays using Annexin-V^+^ DAPI^-^ as a marker in flow cytometry or cleaved caspase 3 as a marker in western blot indicated that UQCRH overexpression enhanced apoptosis in KMRC2 (Fig. 4b, c). Subsequently, we examined the effect of UQCRH overexpression on KMRC2 subcutaneous tumor growth in immunodeficient mice. Consistent with the in vitro result, UQCRH overexpression slowed down the tumor growth in vivo (Fig. 4d). Immunohistochemistry (IHC) confirmed UQCRH overexpression and more pronounced cleaved caspase 3 signals in tumors formed by KMRC2-UQCRH (Fig. 4e).

Probably because lowering UQCRH expression in a cell line with already weakened mitochondrial function would not further affect much of the cellular activities, *UQCRH* knockout did not affect 786O proliferation in vitro (Fig. 4f, g) or as orthotopic tumors in vivo (Fig. 4h). Together with the Seahorse data (Fig. 3d,e), our results indicate that UQCRH is dispensable for metabolic activity and cellular proliferation in 786O.

## Discussion

Our study focused on the clinical relevance and functional impact of *UQCRH* hypermethylation and downregulation in ccRCC and made several interesting observations: (1) Low UQCRH expression in ccRCC is associated with poorer survival, and the loss of UQCRH function in ccRCC is unlikely to be compensated by UQCRHL; (2) DNMT inhibitor decitabine restored UQCRH expression in *UQCRH*-methylated ccRCC cell line KMRC2; (3) enforced UQCRH expression in KMRC2 repolarized mitochondrial membrane potential, increased glycolytic reserve, enhanced basal and ATP-linked oxygen consumption, and shifted cells to an overall less Warburg-like state; (4) UQCRH overexpression in KMRC2 induced higher rates of apoptosis and slowed down in vitro and in vivo tumor growth; (5) *UQCRH* knockout did not influence cellular metabolism or tumor growth in 786O, suggesting that UQCRH loss becomes dispensable for mitochondrial activity and cell proliferative potential once ccRCC cells have entered a Warburg-like state through other molecular changes such as HIF hyperactivation. Taken together, these data provide functional evidence that UQCRH can restrict ccRCC progression through sustaining complex III integrity and mitochondrial function, and that hypermethylation of *UQCRH* promotes the Warburg effect in at least a subset of ccRCC cases.

Among the nucleus-encoded complex III genes, *UQCRH* is particularly interesting in ccRCC. Metabolomics studies on ccRCC show that OXPHOS metabolites do not correlate well with relevant mRNA levels ^7^. There is also a strong uncoupling of OXPHOS mRNA and protein expression, a phenomenon not observed from other cellular pathways^6^. However, through promoter hypermethylation, UQCRH is downregulated consistently at both mRNA and protein levels. This result strongly supports the use of UQCRH downregulation as a new biomarker for mitochondrial dysfunction and poor prognosis in ccRCC, a notion consistent with the recent report^18^. In ccRCC, overall promoter DNA hypermethylation is correlated with higher tumor stage and grade^5^. Many tumor suppressor genes, such as *VHL*, members of the Wnt and TGFβ pathways and pro-apoptotic genes, have been identified to be partially or completely silenced due to hypermethylation in ccRCC^24^. Our study adds *UQCRH* into this list and further supports the idea of targeting ccRCC with DNMT inhibitors (like decitabine) as monotherapy or in combination with other therapies^24^.

Our study demonstrates that *UQCRH* is a putative tumor suppressor gene for ccRCC, based on the effect of UQCRH overexpression on both in vitro metabolism assays as well as in vivo tumor growth. Among the results, the increased apoptosis by UQCRH overexpression is consistent with Okazaki et al., which showed that UQCRH overexpression in murine promyeloid cells accelerated apoptosis through the release of cytochrome c from mitochondria to cytosol^25^. Our results cannot explain the clinical observations that UQCRH is upregulated in lung and liver cancers^20,21^. It is highly possible that the expression pattern and functional contribution of UQCRH to cancer are dependent on the metabolic activity of distinct tumor types as well as the specific mutation landscape and cellular context even for the same tumor type. For example, while ccRCC exhibits classic Warburg effect, lung adenocarcinoma displays high mitochondrial activity and OXPHOS based on both in vivo tracing experiment^8^ and in vivo sensor of mitochondrial membrane potential^26^. As a consequence, UQCRH displays disparate association with different cancers, a phenomenon with implications in cancer prognosis and treatment.

## Methods

### Cell culture

KMRC2 (Japanese Collection of Research Bioresources Cell Bank, JCRB1011) and 786O (American Type Culture Collection, CRL-1932) were cultured in Dulbecco’s Modified Eagle's Medium (GE Healthcare, SH30243.FS) or RPMI 1640 medium (GE Healthcare, SH30027.01), respectively, supplemented with 10% fetal bovine serum (GE Healthcare, SH30396.03) and 1X Penicillin–Streptomycin (Caisson Labs, PSL01) at 37 °C in a humidified incubator with 5% CO_2_.

### Decitabine treatment and western blot

KMRC2 cells were plated and treated with DMSO, 1 μM, 3 μM or 10 μM decitabine for 72 h. Culture medium was changed every 24 h. For western blot, the same procedures we reported^27^ were followed. Primary antibodies included rabbit monoclonal anti-UQCRH (abcam, ab134949) and mouse monoclonal anti-β-actin (Santa Cruz, sc-47778). Full size original blots are shown in Supplementary Fig. 2.

### UQCRH overexpression and CRISPR/Cas9 knockout for stable cell lines

For ectopic overexpression, the human UQCRH ORF in pcDNA3.1^+^/C-(K)DYK backbone (Genscript, OHu28762) was extracted with EndoFree Plasmid Maxi Kit (QIAGEN). The plasmid or control vector was transfected into KMRC2 with Lipofectamine 2000 (Invitrogen). After 96 h, stable cells were selected using 1.2 mg/ml G418 for 14 days. The stable overexpression sublines were cultured in the presence of 1.2 mg/ml G418. For knockout, lentivirus encoding scramble sgRNA (ABM, K010) or sgRNA specific for human *UQCRH* (ABM, K2593405, sequence CTTCCCACTGACTTACGCAA) in all-in-one vector was packaged in 293T and used to infect 786O for 3 repeats. Stable cells were selected and maintained in 2 μg/ml puromycin.

### Mitochondrial membrane potential analysis

Cells were stained with 1X JC-1 (5,5′,6,6′-tetrachloro-1,1′,3,3′-tetraethylbenzimidazolylcarbocyanine iodide) in 1X assay buffer according to manufacturer instructions (Cell Technology, JC100). Red and green fluorescence were detected and quantified on a BD LSRFortessa X-20 flow cytometer. To quantify Mean Fluorescence Intensity (MFI) for the green signals, the MFI of FITC channel was normalized to FSC-A signals.

### Seahorse assays

The Seahorse Extracellular Flux XF96 Analyzer (Agilent) was used to measure oxygen consumption rate (OCR) and extracellular acidification rate (ECAR) in two assays, Cell Mitochondrial Stress Test (Agilent) and Glycolysis Stress Test (Agilent), following manufacturer instructions. Specifically, 2 × 10^4^ KMRC2 or 786O cells per well were plated in quadruplicates in a 96-well XF culture plate in DMEM XF assay media (Agilent) supplemented with proper concentrations of glucose, sodium pyruvate and L-glutamine. For Mitochondrial Stress Test, oligomycin at 1.5 µM, FCCP at 1 µM and Rotenone/Antimycin A at 0.5 µM were optimized concentrations. For Glycolysis Stress Test, saturating glucose solution at 25 mM, oligomycin at 1 µM and 2-deoxy-glucose (2-DG) at 50 mM were optimized concentrations. After the assay, OCR and ECAR readings were normalized by crystal violet absorbance readings, which are a proxy for total cell content in each well. Cells were fixed and stained with 0.2% crystal violet/25% methanol solution for 10 min at room temperature. Excess stains were washed with PBS and ddH_2_O. SDS (1%) was added to solubilize crystal violet stain, following by shaking the plate for 5 min at 350 rpm on MixMate (Eppendorf). Finally, the absorbance was read at 570 nm subtracting the background absorbance at 690 nm.

### Reactive oxygen species (ROS) measurement

Cells at 1.3 × 10^4^ per well were seeded into white 96-well plate and incubated overnight. DCFDA (2′,7′-dichlorofluorescin diacetate) was added at 10 μM, and the plate was incubated for another 2.5 h. Fluorescence was measured on a plate reader at an excitation wavelength of 495 nm and an emission wavelength of 529 nm.

### Apoptosis assays

For flow cytometry, cells were stained with APC-conjugated Annexin V (Tonbo Biosciences, 20-6409-T025) and DAPI in 1X Annexin V binding buffer according to manufacturer instructions (R&D Systems, 4830-01-K). Fluorescence was detected and quantified on a BD LSRFortessa X-20 flow cytometer. Cells treated with 1 µM staurosporine for 4 h were used as the positive control for apoptosis. For western blot and IHC detection of apoptosis, rabbit anti-cleaved caspase 3 antibody (CST, 9661) was used.

### In vitro proliferation assays

For KMRC2, cells at 3 × 10^4^ per well were seeded into clear 6-well plate and incubated for 7 days. Cells were fixed by 4% paraformaldehyde for 15 min at room temperature and permeabilized by 90% ice-cold methanol for 30 min on ice. Cell nuclei were then stained with 1 µg/mL propidium iodide for 30 min and imaged with the 20 × objective of the IncuCyte ZOOM imaging system (Essen BioScience). Cell numbers were quantified with a custom R program. For 786O proliferation curves, cells at 1 × 10^5^ cells per well were seeded into 12-well plate. After 24 h, the plate was placed in IncuCyte ZOOM (Essen BioScience) and imaged every 2 h until confluency. Cell confluency per well was calculated, and a proliferation curve was plotted by the IncuCyte software. For the colony formation assay, 786O sublines at 400 cells per well were seeded into 6-cm plates and incubated for 7 days. Cells were stained with crystal violet, and total colony number per well was counted.

### In vivo tumor growth assays

For each KMRC2 subline, 2 × 10^6^ cells were inoculated subcutaneously at both flanks into 6-week-old Rag1 knockout male mice (Jackson Laboratories, 003145). Tumors were measured by calipers. Tumor volume was calculated by the formula 0.5 × (Length × Width^2^). For 786O sublines, orthotopic xenografts were generated by injecting 10^6^ cells into the lower pole of the renal parenchyma of female NCr nude mice (Taconic). Mice were sacrificed at 4 weeks post injection to harvest the kidneys for histology. Contour of the kidney and the tumor area on H&E staining section were drawn with ImageJ to measure surface area for the calculation of tumor area percentage. All animals were maintained in pathogen-free conditions, and all manipulations were approved by the Institutional Animal Care and Use Committee (IACUC) at the University of Notre Dame. We confirm that all experiments were performed in accordance with relevant guidelines and regulations.

### Statistical analysis

Numbers of biological replicates (n) for the experiments were denoted in figures or legends. Methods of statistical tests were indicated in figures or legends. *P* < 0.05 was considered statistically significant.

## Supplementary information

Supplementary Information.

## Data Availability

The data that support the findings of this study are available from the corresponding author upon reasonable request.
