# Supplementary material for: UQCRH downregulation promotes Warburg effect in renal cell carcinoma cells
Source: Sci Rep. 2020 Sep 14;10:15021. doi: 10.1038/s41598-020-72107-2 (PMC7490363; doi:10.1038/s41598-020-72107-2)
Supplement: Supplementary file 1 — Supplementary Information. [file 41598_2020_72107_MOESM1_ESM.pdf]

Supplementary Information

UQCRH Downregulation Promotes Warburg Effect in Renal Cell Carcinoma Cells

Yanting Luo , Louise Medina Bengtsson, Xuechun Wang, Tianhe Huang, Guoqiang Liu, Sean Murphy, Caiqin Wang, John Koren III, Zachary Schafer, Xin Lu

Supplementary Figure 1

Supplementary Figure 2

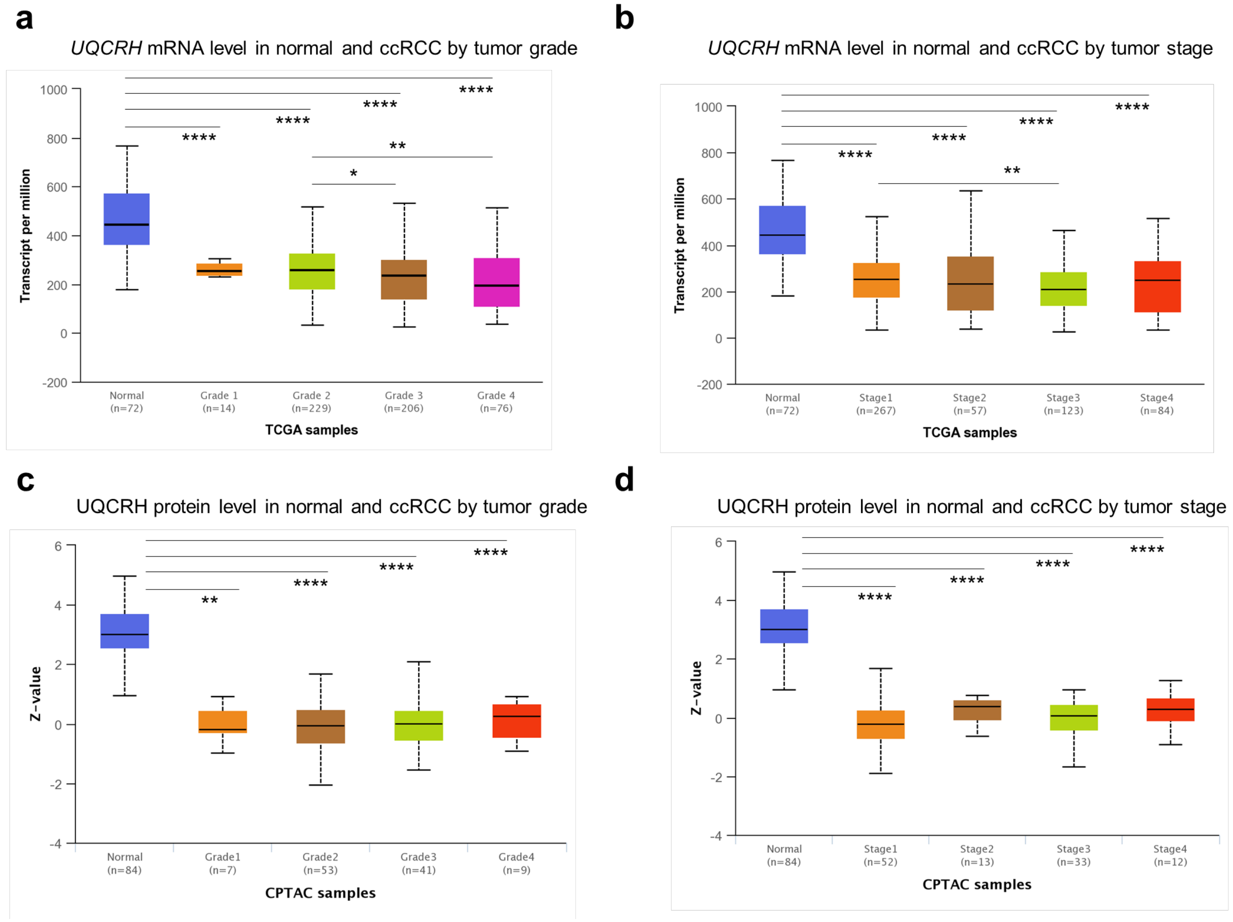

Supplementary Figure 1. UQCRH expression analysis in ccRCC. (**a-b**) *UQCRH* transcript level in normal tissue and ccRCC of progressive grades or stages, based on TCGA data and analyzed with UALCAN. (**c-d**) UQCRH protein level in normal and ccRCC of progressive grades or stages, based on CPTAC data and analyzed with UALCAN. Definition for grades: Grade 1, well differentiated (low grade); Grade 2, moderately differentiated (intermediate grade); Grade 3, poorly differentiated (high grade); Grade 4, undifferentiated (high grade). *P<0.05, **P<0.01, ****P<0.0001, unpaired t-test.

Fig. 2c

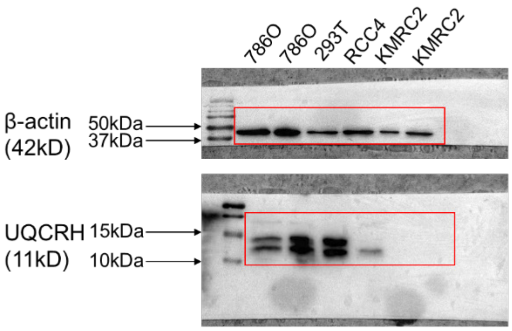

Fig. 2d

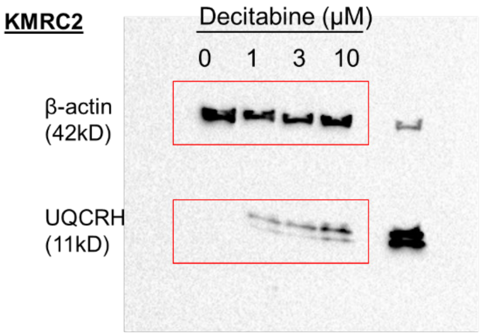

Fig .2e

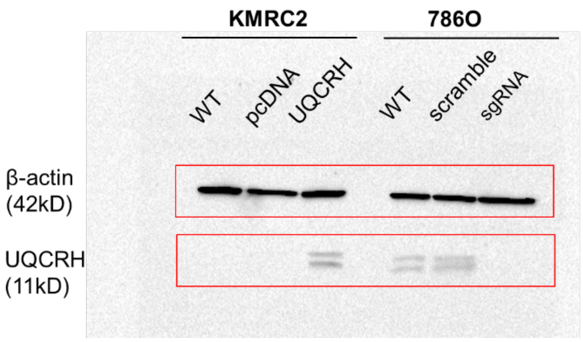

Fig .4c

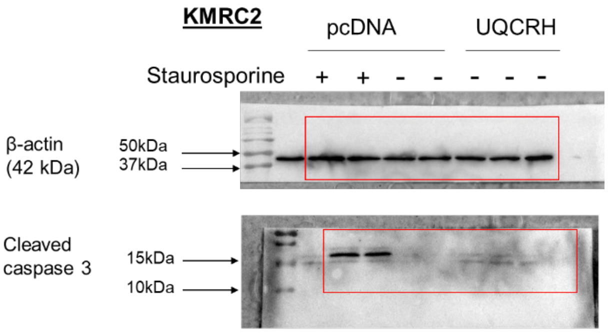

Supplemental Figure 2. Original unprocessed blots for the western blot results shown in main figures. Red rectangles indicate the areas of the blots that are cropped to show in main figure panels.
